# Baseline Systemic Inflammation Response Index and Clinical Outcomes in Heavily Pretreated Platinum-Resistant Ovarian Cancer Treated with Pembrolizumab Monotherapy

**DOI:** 10.3390/cancers18182905

**Published:** 2026-09-08

**Authors:** Seongyun Lim, Yurimi Lee, Heeeun Ha, Young Eun Chung, Jun-Hyeong Seo, Chel-Hun Choi, Tae-Joong Kim, Jeong-Won Lee, Yoo-Young Lee

**Affiliations:** 1 Gynecologic Cancer Center, Department of Obstetrics and Gynecology, Samsung Medical Center, Sungkyunkwan University School of Medicine, 81, Irwon-ro, Gangnam-gu, Seoul 06351, Republic of Korea; 2 Department of Pathology and Translational Genomics, Samsung Medical Center, Sungkyunkwan University School of Medicine, Seoul 06351, Republic of Korea

**Keywords:** ovarian cancer, platinum-resistant, systemic inflammation response index, pembrolizumab, immune checkpoint inhibitor

## Abstract

**Objective:** Immune checkpoint inhibitor monotherapy has shown limited efficacy in platinum-resistant ovarian cancer (PROC), although a small subset of patients experiences durable disease control. We evaluated the association between baseline systemic inflammatory markers and the clinical outcomes of pembrolizumab monotherapy. **Materials and Methods:** We included 95 patients with PROC who received pembrolizumab monotherapy at Samsung Medical Center between 2018 and 2023. Durable disease control was defined as a progression-free survival (PFS) ≥ 6 months. The systemic inflammation response index (SIRI), neutrophil-to-lymphocyte ratio (NLR), and systemic immune-inflammation index (SII) were calculated from the pretreatment complete blood count. Associations with the PFS and overall survival (OS) were evaluated using multivariable Cox regression. **Results:** Among 94 patients evaluable for 6-month durable disease control, 10 (10.6%) achieved this endpoint. The patients with durable disease control had a lower baseline SIRI than those without durable disease control (median, 0.7 vs. 1.6; *p* = 0.019). In multivariable analyses, a higher log-transformed SIRI was associated with a shorter PFS (HR, 1.44; 95% CI, 1.13–1.83; *p* = 0.003) and OS (HR, 2.02; 95% CI, 1.53–2.67; *p* < 0.001). **Conclusions:** The baseline SIRI was associated with the PFS and OS in heavily pretreated patients with PROC receiving pembrolizumab monotherapy, and a lower SIRI was associated with durable disease control. The SIRI may provide readily available prognostic information in this setting; however, the data-derived cutoff requires prospective external validation and should not be interpreted as a predictor of the pembrolizumab-specific benefit.

## Simple Summary

Platinum-resistant ovarian cancer is difficult to treat, and the immunotherapy drug pembrolizumab provides long-lasting cancer control in only a small proportion of patients. We investigated whether routine blood tests taken before treatment could provide information about which patients tend to have better outcomes. We focused on inflammatory blood-based markers and found that patients with a lower systemic inflammation response index (SIRI) tended to have longer cancer control and survival. SIRI can be calculated easily from standard blood cell counts and does not require additional testing. However, this study was retrospective and conducted at a single institution, and it cannot determine whether SIRI specifically predicts the effect of pembrolizumab. Larger studies are needed before SIRI can be used to guide treatment decisions.

## 1. Introduction

Epithelial ovarian cancer remains one of the most lethal gynecological malignancies. Despite an initial sensitivity to platinum-based chemotherapy, most patients eventually develop platinum-resistant ovarian cancer (PROC), characterized by limited treatment options and a median overall survival (OS) of approximately 12–18 months [1].

Immune checkpoint inhibitors (ICIs), including pembrolizumab, have improved the clinical outcomes across several solid tumors. In PROC, however, ICI monotherapy has shown only a modest overall efficacy [2,3,4]. In the KEYNOTE-100 trial, pembrolizumab monotherapy resulted in an objective response rate (ORR) of approximately 8% and a median progression-free survival (PFS) of 2.1 months [5]. Notably, among responders in the heavily pretreated cohort (4–6 prior lines), the median duration of response was 23.6 months, suggesting that, although responses are uncommon, they can be prolonged.

More recently, the KEYNOTE-B96 trial demonstrated that adding pembrolizumab to weekly paclitaxel, with or without bevacizumab, improved the PFS and OS in patients with PROC who had received one or two prior systemic regimens [6]. However, these findings may not be directly applicable to heavily pretreated patients with cumulative treatment-related toxicity, frailty, a declining performance status, or a limited tolerance for further cytotoxic chemotherapy. In such patients, pembrolizumab monotherapy has been used as a less intensive treatment option, despite its modest overall efficacy. Therefore, identifying clinical factors associated with the outcomes after pembrolizumab monotherapy remains relevant in this selected population.

Systemic inflammatory markers derived from a routine complete blood count (CBC), including the neutrophil-to-lymphocyte ratio (NLR), systemic immune-inflammation index (SII), and systemic inflammation response index (SIRI), have gained increasing recognition as prognostic tools in ICI-treated malignancies [7,8,9,10,11]. These indices integrate varying degrees of neutrophilia, monocytosis, and relative lymphopenia, and may therefore reflect both systemic inflammation and host-immune competence. Because the activity of the PD-1 blockade depends in part on functional antitumor effector immunity, an unfavorable systemic inflammatory state before treatment may be associated with poorer outcomes during ICI therapy [12].

In this study, we investigated whether baseline CBC-derived systemic inflammatory markers were associated with the clinical outcomes in heavily pretreated patients with PROC receiving pembrolizumab monotherapy. We additionally explored their association with durable disease control and performed exploratory threshold analyses.

## 2. Materials and Methods

This was a single-center retrospective cohort study conducted at Samsung Medical Center, Seoul, South Korea. Patients were eligible if they had histologically confirmed epithelial ovarian, fallopian tube, or primary peritoneal cancer with platinum-resistant disease—defined as radiographic progression within 6 months of completing the last platinum-based chemotherapy—and received pembrolizumab monotherapy between July 2018 and December 2023. During the study period, institutional approval for off-label pembrolizumab generally required PD-L1 positivity (combined positive score [CPS] ≥ 1 or tumor proportion score (TPS) ≥ 1% by PD-L1 IHC 22C3 pharmDx assay [Agilent Technologies, Inc., Santa Clara, CA, USA]) or deficient mismatch repair (dMMR), defined as the loss of at least one of MLH1, MSH2, MSH6, or PMS2 by immunohistochemistry.

Patients were excluded for missing inflammatory marker data, combination pembrolizumab therapy, a concurrent malignancy, an indeterminate platinum resistance status, an age < 18 years, or a known active autoimmune or hematologic disease. Treatment selection was made in routine clinical practice at the discretion of the treating physician and patient; specific patient-level reasons for selecting pembrolizumab monotherapy were not systematically captured as categorical variables.

The study was approved by the institutional review board of Samsung Medical Center (No. 2025-05-013-001). Individual informed consent was waived given the retrospective design.

Pembrolizumab, 200 mg, was administered intravenously every 3 weeks, without concurrent cytotoxic chemotherapy. Dose reductions were not used, whereas information on temporary dose delays or treatment interruptions was not systematically collected for the present analysis. A radiologic assessment using CT or MRI was performed every 3 cycles; additional imaging was conducted after 2 cycles when rising serum CA-125 levels or changes in clinical symptoms prompted an earlier evaluation. The tumor response was assessed as per the Response Evaluation Criteria in Solid Tumors (RECIST) version 1.1.

The PFS was defined as the time from pembrolizumab initiation to radiological progression, or death from any cause, whichever occurred first. Patients without a PFS event were censored at the date of their last disease assessment. The OS was defined as the time from pembrolizumab initiation to death from any cause, with patients alive at the data cutoff censored at the date they were last known to be alive. The data cutoff was 31 December 2024. Patients were categorized according to the presence or absence of durable disease control, defined as a progression-free survival (PFS) of ≥6 months, consistent with the established endpoint in ICI trials [13].

Clinical and pathological data were extracted from electronic medical records, including the age, Eastern Cooperative Oncology Group (ECOG) performance status, International Federation of Gynecology and Obstetrics (FIGO) 2017 stage at the initial diagnosis, histological type, tumor grade, completeness of primary surgery (residual disease status), number of prior systemic chemotherapy lines, prior bevacizumab and PARP inhibitor use, PD-L1 expression and dMMR status, BRCA mutation status, serum albumin and serum CA-125.

The abdominopelvic CT examination obtained closest to pembrolizumab initiation and within the preceding 90 days was reviewed for radiographically evident ascites and definite active peritoneal involvement. Peritoneal involvement was defined as explicit radiographic evidence of active peritoneal seeding, carcinomatosis, or metastatic peritoneal thickening. The interval between imaging and pembrolizumab initiation was recorded. Post-progression treatment was also reviewed among patients with documented disease progression.

To further characterize the preceding treatment trajectory, we calculated the interval from the initiation of the first systemic treatment to pembrolizumab initiation for each patient. This interval was considered a measure of the overall prior treatment-course duration rather than the regimen-specific duration of the response.

Exact PD-L1 CPS scores were available for 59 patients; the categorical PD-L1 status was abstracted when exact CPS values were unavailable. MMR testing was available for all patients with durable disease control, but was performed selectively among patients without durable disease control. Therefore, the MMR status was summarized descriptively because testing was incomplete and non-random.

The baseline CBC values were retrospectively abstracted from routine laboratory tests performed as part of standard clinical care within 1–2 days before the first pembrolizumab administration. Inflammatory markers were calculated as follows:

- Systemic immune inflammation index (SII): (platelet count × neutrophil count) ÷ lymphocyte count
- Systemic inflammation response index (SIRI): (neutrophil count × monocyte count) ÷ lymphocyte count
- Neutrophil-to-lymphocyte ratio (NLR): neutrophil count ÷ lymphocyte count

Because all three markers showed right-skewed distributions, they were natural-log-transformed for the regression analyses (Figure S1).

The baseline characteristics were compared between patients with and without durable disease control using Fisher’s exact test for categorical variables and Student’s *t*-test or the Mann–Whitney U test for continuous variables, as appropriate.

Associations between baseline inflammatory markers and the PFS and OS were first evaluated using univariable Cox proportional hazards models. Multivariable Cox models were adjusted for the age, ECOG status, number of prior treatment lines, and serum albumin, with each inflammatory marker evaluated in a separate model to avoid collinearity among related CBC-derived indices. Proportional hazards assumptions were assessed using Schoenfeld residuals.

Subgroup and sensitivity analyses were performed to assess the robustness of the findings. These included restriction to patients with serous histology, an additional adjustment for the baseline disease extent and prior bevacizumab/PARP inhibitor exposure, and the replacement of prior treatment lines with the duration of the preceding treatment course. The prior treatment-course duration was strongly correlated with the number of prior treatment lines (Spearman ρ = 0.85, *p* < 0.001); therefore, the two variables were not entered simultaneously in the same multivariable model.

As an exploratory analysis of durable disease control, receiver operating characteristic (ROC) curves were constructed for each log-transformed inflammatory marker. The areas under the curves (AUCs) were estimated and compared using the DeLong method, and data-derived thresholds were identified using the Youden index. Because both marker selection and threshold derivation were performed within the same cohort, threshold-based analyses were considered exploratory and hypothesis-generating. A sensitivity ROC analysis using a PFS ≥ 12 months as a more stringent definition of durable disease control was also performed.

Among the evaluated inflammatory markers, the SIRI was prioritized for subsequent focused analyses because it showed the strongest association with durable disease control and the numerically highest discriminatory performance. Subsequent sensitivity analyses therefore focused on the SIRI. Using the exploratory SIRI threshold, the PFS and OS were estimated using the Kaplan–Meier method and compared using the log-rank test. The restricted mean survival time (RMST) was calculated at 12 months for the PFS and 24 months for the OS.

All of the statistical tests were two-sided, and *p*-values < 0.05 were considered statistically significant. Given the exploratory nature of the marker and threshold analyses, no adjustment for multiple comparisons was performed. The analyses were conducted using R version 4.4.1 (R Foundation for Statistical Computing, Vienna, Austria).

## 3. Results

**Figure 1:**
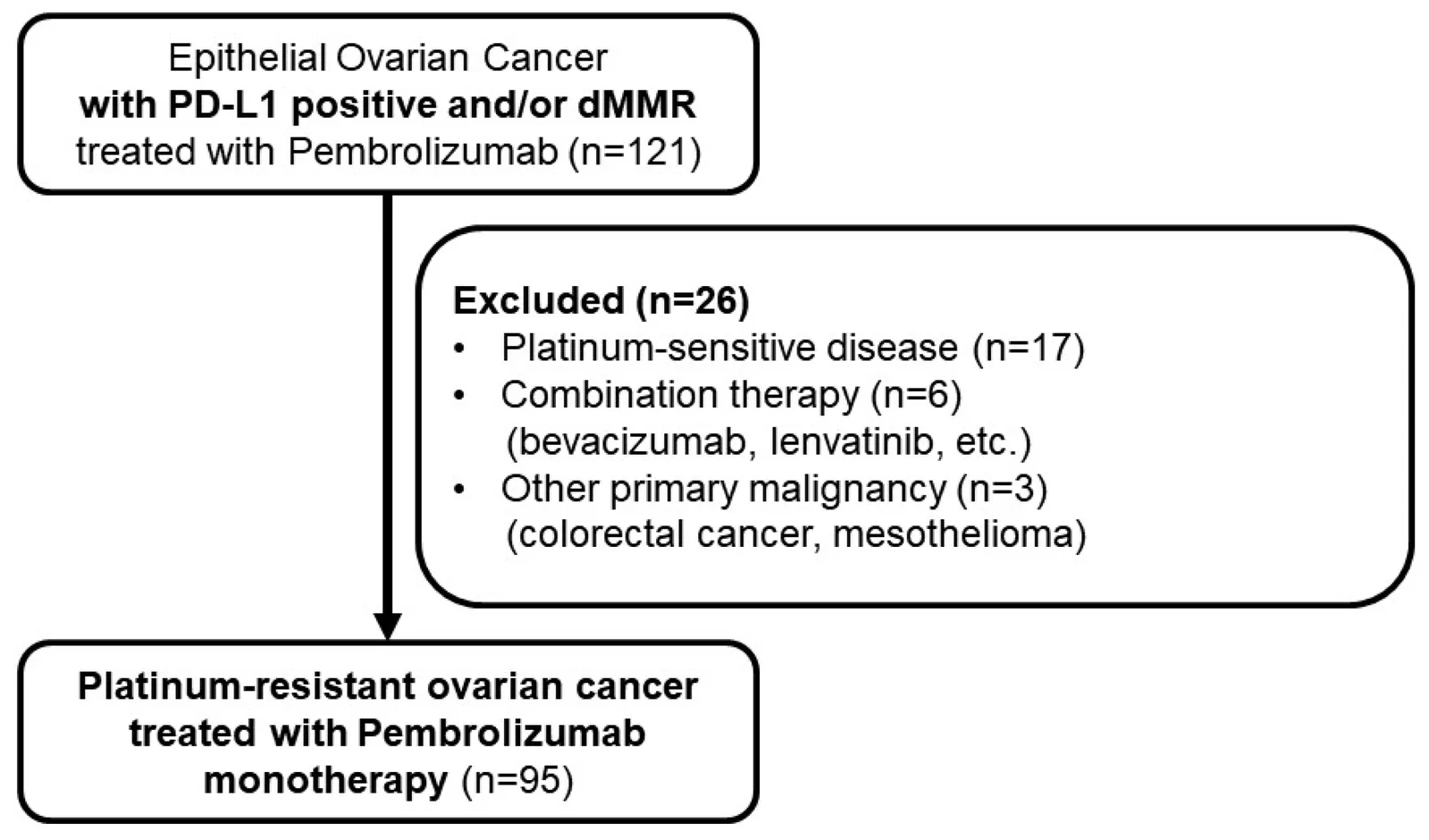
Flow diagram of the study population selection process. Out of 121 patients with epithelial ovarian cancer that were treated with pembrolizumab and screened for eligibility, 26 were excluded. The reasons for exclusion included platinum-sensitive disease (*n* = 17), the receipt of combination therapy (e.g., bevacizumab, lenvatinib) (*n* = 6), and a diagnosis of other primary malignancies (*n* = 3). Patients with missing pretreatment inflammatory marker data, an indeterminate platinum resistance status, an age < 18 years, or active autoimmune or hematologic disease met the prespecified exclusion criteria, but no additional exclusions were identified. Ultimately, 95 patients with platinum-resistant ovarian cancer treated with pembrolizumab monotherapy were included in the final analysis.

Of 121 patients with PROC who received pembrolizumab, 95 met the inclusion criteria after excluding those with platinum-sensitive disease (*n* = 17), combination therapy (*n* = 6), or concurrent malignancies (*n* = 3) (Figure 1).

The baseline characteristics are summarized in Table 1. The final cohort was heavily pretreated, with a median of four prior lines of systemic therapy, and 66 patients (69.5%) had previously received bevacizumab. Twenty-three patients (24.2%) had an ECOG performance status ≥ 2. The median follow-up duration was 54 months (range, 15–77 months).

The median interval between CT acquisition and pembrolizumab initiation was 11 days (IQR, 5–21; range, 0–89 days), and all scans were obtained within 90 days before treatment initiation. Radiographically evident ascites was present in 29 patients (30.5%), and definite active peritoneal involvement was identified in 69 patients (72.6%). The median time from the initiation of the first systemic treatment to pembrolizumab was 29.6 months (IQR, 16.0–52.9 months).

In the overall cohort, the median PFS was 2 months and the median OS was 7 months. The ORR was 8.4% (*n* = 8), and the disease control rate (DCR) was 25.3% (*n* = 24). The median duration of response was 12 months (interquartile range [IQR], 5.0–41.5). Among the 94 patients evaluable for 6-month durable disease control, 10 (10.6%) achieved durable disease control. Their median PFS was 14.0 months (95% CI, 6.0–not reached) and three patients remained progression-free at the last follow-up. One patient was censored before 6 months without progression or death and could not be classified according to this binary endpoint.

Patients with durable disease control had a lower median SIRI than those without durable disease control (0.7 vs. 1.6, *p* = 0.019). The NLR was also numerically lower (1.9 vs. 3.0, *p* = 0.061), whereas the SII did not differ significantly (467 vs. 765, *p* = 0.116). The interval from the initiation of the first systemic treatment to pembrolizumab was numerically longer among patients with durable disease control (48.9 vs. 25.8 months, *p* = 0.074). No significant differences were observed in other major clinicopathologic or prior-treatment characteristics.

**Figure 2:**
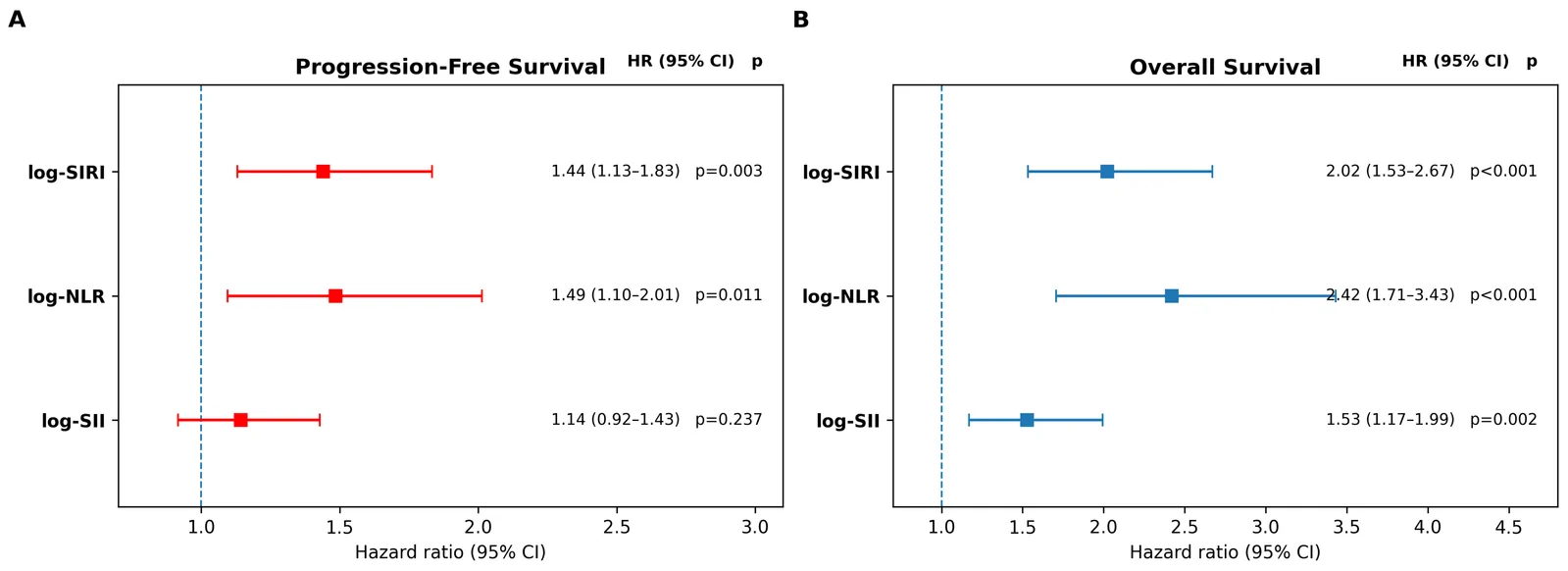
(**A**) Progression-Free Survival. (**B**) Overall Survival. Forest plot of multivariable Cox proportional hazards regression analysis for inflammatory markers associated with survival outcomes. The plot displays the hazard ratios (HRs) and 95% confidence intervals (CIs) for the progression-free survival (PFS) and overall survival (OS) associated with log-transformed inflammatory markers. Each marker was analyzed in a separate multivariable model adjusted for the age, line of treatment, serum albumin, and ECOG performance status. The vertical dashed line represents an HR of 1.0 (no effect).

In the univariable analysis, a higher log-SIRI was associated with a shorter PFS (HR: 1.56, 95% CI: 1.24–1.97, *p* < 0.001) and OS (HR: 2.18, 95% CI: 1.68–2.83, *p* < 0.001) (Table S1). In the multivariable models adjusted for the age, serum albumin, number of prior treatment lines, and ECOG performance status, the log-SIRI remained associated with both the PFS (HR: 1.44, 95% CI: 1.13–1.83, *p* = 0.003) and the OS (HR: 2.02, 95% CI: 1.53–2.67, *p* < 0.001). The log-NLR showed similar associations with the PFS (HR: 1.49, 95% CI: 1.10–2.01, *p* = 0.011) and the OS (HR: 2.42, 95% CI: 1.71–3.43, *p* < 0.001), whereas the log-SII was associated with the OS (HR: 1.53, 95% CI: 1.17–1.99, *p* = 0.002), but not the PFS (HR: 1.14, 95% CI: 0.92–1.43, *p* = 0.237) (Table 2 and Figure 2). The serum albumin was independently associated with the OS (HR: 0.40, 95% CI: 0.26–0.60, *p* < 0.001). The proportional hazards assumption was satisfied for the PFS model (global *p* = 0.260).

The association of the log-SIRI with the survival outcomes remained consistent across multiple sensitivity analyses (Table S2). In particular, an additional adjustment for the baseline disease extent, including ascites, definite peritoneal involvement, and CA-125, did not materially alter the association. Similar findings were observed in analyses restricted to serous histology and after accounting for prior bevacizumab/PARP inhibitor exposure, the prior treatment-course duration, and the PD-L1 status.

In exploratory ROC analyses among the 94 patients evaluable for 6-month durable disease control, the log-SIRI showed the highest numerical AUC (0.727, 95% CI: 0.577–0.878) compared with the log-NLR (0.682, 95% CI: 0.513–0.851) and log-SII (0.653, 95% CI: 0.481–0.825), although pairwise AUC differences were not statistically significant (Figure S2, Table S3). The Youden-derived SIRI threshold was 1.15, corresponding to a sensitivity of 80.0%, a specificity of 64.3%, and a negative predictive value of 96.4%. The same threshold was obtained when durable disease control was defined more stringently as a PFS ≥ 12 months, with an AUC of 0.733 (95% CI 0.575–0.891) (Table S4). Because both marker prioritization and threshold derivation were performed within the same cohort, these threshold-based analyses were considered exploratory.

**Figure 3:**
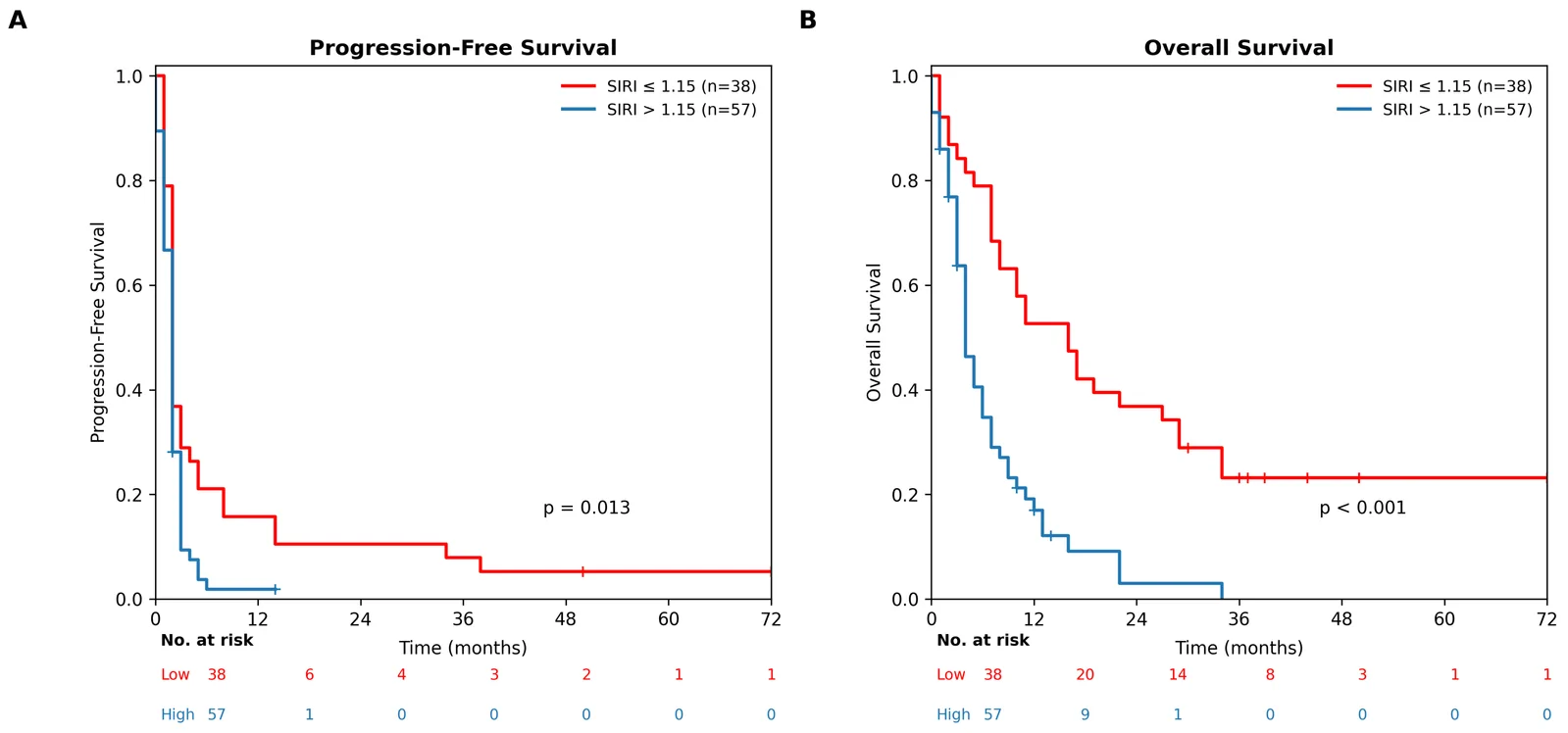
(**A**) Progression-Free Survival. (**B**) Overall Survival. Kaplan-Meier estimates of the progression-free survival (PFS) and overall survival (OS) stratified by the baseline SIRI. Patients were dichotomized into low-SIRI (≤1.15, *n* = 38) and high-SIRI (>1.15, *n* = 57) groups based on the Youden-optimal cutoff. *p*-values were calculated using the log-rank test. Tick marks indicate censored observations. Abbreviations: SIRI, systemic inflammation response index; PFS, progression-free survival; OS, overall survival.

Using the exploratory SIRI threshold, 38 patients (40.0%) were classified as a low SIRI (≤1.15) and 57 (60.0%) as a high SIRI (>1.15) (Tables S5 and S6). Although the median PFS was 2 months in both groups, the PFS curves differed significantly (log-rank *p* = 0.013, Figure 3), with higher landmark PFS rates and longer 12-month RMST in the low-SIRI group. The median OS was 16 versus 4 months (log-rank *p* < 0.001). The ORR was higher in the low-SIRI group than in the high-SIRI group (18.4% vs. 1.8%, *p* = 0.006), whereas the DCR did not differ significantly (34.2% vs. 19.3%, *p* = 0.148).

Finally, among the 88 patients with documented disease progression, subsequent systemic therapy was more frequently administered in the low-SIRI group than in the high-SIRI group (68.6% vs. 39.6%, *p* = 0.010). The number of subsequent treatment lines was also greater in the low-SIRI group (median, 2 [IQR, 0–3] vs. 0 [0–1]; *p* = 0.002).

## 4. Discussion

In this cohort of heavily pretreated patients with PROC receiving pembrolizumab monotherapy, a lower systemic inflammatory burden was associated with durable disease control. The baseline SIRI was associated with both the PFS and OS after an adjustment for clinical factors, and these associations remained consistent in sensitivity analyses accounting for the baseline disease extent, prior treatment exposure, prior treatment-course duration, and PD-L1 expression. Among the inflammatory indices evaluated, the SIRI showed the highest numerical discrimination for durable disease control, although it was not statistically superior to the NLR or SII.

The SIRI was initially developed as a prognostic marker in pancreatic cancer and has subsequently been associated with adverse survival outcomes across a broad range of solid tumors [14,15,16,17,18] In our cohort, the NLR was also associated with both the PFS and OS; however, the SIRI showed the highest numerical discrimination for durable disease control and was therefore emphasized in subsequent exploratory analyses, although its AUC was not significantly higher than those of the NLR or SII. Consistent with our findings, a higher SIRI has been associated with poorer outcomes in anti-PD-1-treated non-small cell lung cancer [19], while pretreatment NLR has also been associated with outcomes in ICI-treated endometrial, urothelial, and cervical cancers and, more recently, in a real-world cohort of 94 patients with gynecologic malignancies receiving pembrolizumab [20,21,22,23]. These observations support the broader prognostic relevance of the systemic inflammatory status during ICI treatment. However, the thresholds used for these indices have varied across tumor types and study populations, and the data-derived SIRI cutoff identified in our cohort should therefore not be extrapolated to other settings without external validation.

Biologically, the SIRI integrates the neutrophil, monocyte, and lymphocyte counts and may reflect the balance between systemic inflammation, myeloid-mediated immunosuppression, and lymphocyte-dependent antitumor immunity. An unfavorable systemic inflammatory state may therefore be associated with poorer outcomes during a PD-1 blockade [24,25]. However, the SIRI may also reflect the tumor burden, nutritional status, occult inflammation, or general physiologic reserve. Accordingly, our single-arm study cannot establish that the SIRI specifically reflects pembrolizumab sensitivity. The OS association should also be interpreted in the context of greater post-progression treatment exposure among patients with a low SIRI.

The overall efficacy observed in our cohort was consistent with KEYNOTE-100, with an ORR of 8.4% and a median PFS of 2 months [5]. Nevertheless, a small subgroup experienced prolonged disease control: 10 patients (10.5%) remained progression-free for at least 6 months, with a median PFS of 14 months. This long-tail pattern is qualitatively consistent with the long-tail survival pattern of ICI therapy [26,27] and KEYNOTE-100, in which responses were uncommon, but could be prolonged, particularly among heavily pretreated patients.

The treatment landscape of PROC has evolved with KEYNOTE-B96, which demonstrated benefit from adding pembrolizumab to weekly paclitaxel, with or without bevacizumab, in patients with fewer prior treatment lines. In contrast, our cohort received a median of four prior systemic regimens and approximately one-quarter had ECOG ≥ 2. Thus, our findings should not be interpreted as supporting pembrolizumab monotherapy over combination therapy in eligible patients, but rather as describing a more heavily pretreated population in whom further cytotoxic therapy may be less feasible.

Despite extensive biomarker research within KEYNOTE-100, no molecular markers, including the tumor mutational burden, T-cell inflamed gene expression profiles, and CD8+ tumor-infiltrating lymphocyte density, can successfully predict the durable benefit [28]. In this context, CBC-derived inflammatory indices are attractive because they are inexpensive, routinely available, and easy to obtain. However, because no non-ICI comparator was included, the present study demonstrates a prognostic association rather than pembrolizumab-specific predictive value. Future studies incorporating external validation and an appropriate non-ICI comparator are needed to determine whether the SIRI has treatment-specific predictive utility.

Several limitations must be acknowledged. First, this was a single-center retrospective study with a relatively small sample size, including only 10 patients with durable disease control, limiting the precision of the subgroup and cutoff-based analyses. Although the SIRI remained associated with the outcomes after a multivariable adjustment and sensitivity analyses, residual confounding by the general host condition, the nutritional status, and the disease burden cannot be excluded. Especially because ascites, peritoneal involvement, and CA-125 do not fully quantify the total tumor burden, residual confounding by disease burden remains possible. Systematic screening for an acute infection or subclinical inflammation at the time of CBC sampling was not performed. In addition, the SIRI cutoff was derived and evaluated within the same cohort, and therefore requires external validation. The MMR status was available in only 48 patients and was assessed non-randomly, with testing being more complete among patients with durable disease control. Therefore, MMR-related associations could not be reliably evaluated or excluded as potential confounding. Also, because no non-ICI comparator was included, the present study cannot distinguish a general prognostic association from pembrolizumab-specific predictive value. Finally, the treatment selection and post-progression therapy may have introduced additional selection and survival-related biases, limiting the generalizability of the findings. Off-label pembrolizumab use may introduce socioeconomic selection bias toward patients with a better functional reserve.

## 5. Conclusions

In conclusion, the baseline SIRI was associated with the PFS and OS in heavily pretreated patients with platinum-resistant ovarian cancer receiving pembrolizumab monotherapy. A lower SIRI was also associated with a greater likelihood of durable disease control. These findings support the prognostic relevance of a systemic inflammatory status in this setting, while the data-derived SIRI threshold requires prospective external validation and should not be interpreted as a predictor of the pembrolizumab-specific benefit.

## Figures and Tables

**Table 1:** Baseline characteristics of patients with recurrent platinum-resistant ovarian cancer treated with pembrolizumab monotherapy.

	Total (*n* = 95)	No Durable Control (PFS < 6 Months) (*n* = 84)	Durable Control (PFS ≥ 6 Months) (*n* = 10)	*p*-Value
Clinical characteristics				
Age, mean (SD) years	56.1 (10.8)	55.6 (10.6)	60.9 (11.9)	0.207
≥65 years—No. (%)	18 (18.9)	15 (17.9%)	3 (30.0%)	0.397
ECOG ≥ 2 (poor)	23 (24.2)	22 (26.2%)	1 (10.0%)	0.442
Pathologic characteristics				
Histologic type—No. (%)				1.000
Serous carcinoma	73 (76.8)	65 (77.4%)	8 (80.0%)	
Clear cell carcinoma	14 (14.7)	12 (14.3%)	1 (10.0%)	
Other ^(a)^	8 (8.5)	7 (8.3%)	1 (10.0%)	
High grade	88 (92.6)	79 (94.0%)	8 (80.0%)	0.160
Prior treatment history				
Optimal surgery	39 (41.1)	34 (40.5%)	5 (50.0%)	0.736
Initial FIGO stage III–IV	87 (91.6)	78 (92.9%)	8 (80.0%)	0.201
Prior treatment lines				0.161
1	4 (4.2)	4 (4.8)	0 (0.0)	
2–3	45 (47.4)	42 (50.0)	2 (20.0)	
≥4	46 (48.4)	38 (45.2)	8 (80.0)	
Prior PARP inhibitor use—No. (%)	16 (16.8)	16 (19.0%)	0 (0.0%)	0.202
Prior bevacizumab use—No. (%)	66 (69.5)	60 (71.4%)	5 (50.0%)	0.275
Time from initiation of first systemic treatment to pembrolizumab, median (IQR) months	29.6 (16.0–52.9)	25.8 (14.9–51.4)	48.9 (32.4–73.1)	0.074
Baseline disease extent				
CA125, median (IQR)—U/mL	310 (89.5–695)	332.5 (89.8–730)	192 (90.5–329)	0.528
Radiographically evident ascites, *n* (%)	29 (30.5)	27 (32.1)	2 (20.0)	0.719
Peritoneal involvement, *n* (%) ^(b)^	69 (72.6)	64 (76.2)	5 (50.0)	0.124
Albumin, median (IQR)—g/dL	4.0 (3.5–4.3)	4.0 (3.4–4.3)	4.3 (4.1–4.5)	0.124
Biomarkers				
PD-L1 CPS—No. (%) ^(c)^	*n* = 59	*n* = 48	*n* = 10	0.451
CPS 0	8 (13.6)	7 (14.6%)	1 (10.0%)	
CPS 1–9	22 (37.3)	19 (39.6%)	2 (20.0%)	
CPS ≥ 10	29 (49.2)	22 (45.8%)	7 (70.0%)	
PD-L1 positive, exact CPS unavailable, *n* (%)	27 (28.4)	27 (32.1)	0 (0.0)	
dMMR (*n* = 48 available) ^(c)^	17 (35.4)	14 (37.8%)	3 (30.0%)	
BRCA Positive (*n* = 87 available) ^(c)^	18 (20.7)	16 (21.1%)	2 (20.0%)	1.000
Inflammatory markers, median (IQR)				
SII	752 (365–1648)	765 (381–1690)	467 (260–866)	0.116
SIRI	1.6 (0.8–3.8)	1.6 (0.8–4.2)	0.7 (0.6–1.1)	0.019
NLR	2.7 (1.6–5.7)	3.0 (1.7–5.9)	1.9 (1.3–3.3)	0.061

^(a)^ Includes mucinous carcinoma, malignant Brenner tumors, or mixed histology. ^(b)^ Peritoneal involvement was defined as explicit radiographic evidence of active peritoneal seeding, carcinomatosis, or metastatic peritoneal thickening on imaging obtained closest to pembrolizumab initiation and within the preceding 90 days. Equivocal findings, resolved prior peritoneal disease, and isolated nodal disease were not classified as positive. ^(c)^ Percentages and *p*-values for the PD-L1/CPS, dMMR, and BRCA mutation status were calculated among patients with available data only (CPS *n* = 59, dMMR *n* = 48, BRCA *n* = 87). Patients with PD-L1 positivity confirmed, but without an exact CPS score (*n* = 27), are listed separately; the percentages for this category are relative to the total cohort (*n* = 95). MMR testing was performed in all patients with durable disease control and selectively in non-responders at the treating physician’s discretion. Values are presented as the mean (SD), median (IQR), or number (%). Durable disease control was defined as a PFS ≥ 6 months. One patient who was censored before 6 months without progression or death was excluded from the analyses requiring the binary classification of durable disease control; therefore, the between-group comparisons included 94 evaluable patients. *p*-values were calculated using Student’s *t*-test for normally distributed continuous variables, the Mann–Whitney U test for non-normally distributed continuous variables, and Fisher’s exact test for categorical variables. Statistical significance was defined as *p* < 0.05. Abbreviations: SD, standard deviation; IQR, interquartile range; ECOG, Eastern Cooperative Oncology Group; FIGO, International Federation of Gynecology and Obstetrics; CPS, combined positive score; PD-L1, programmed death-ligand 1; dMMR, mismatch repair deficiency; BRCA, breast cancer susceptibility gene; PARP, poly(ADP-ribose) polymerase; SII, systemic immune-inflammation index; SIRI, systemic inflammation response index; NLR, neutrophil-to-lymphocyte ratio.

**Table 2:** Multivariable Cox proportional hazards analysis of inflammatory markers and clinical factors associated with survival outcomes.

Variable	PFS HR (95% CI)	*p*-Value	OS HR (95% CI)	*p*-Value
(A) Comparative Multivariable Analysis				
log-SIRI	1.44 (1.13–1.83)	0.003	2.02 (1.53–2.67)	<0.001
log-NLR	1.49 (1.10–2.01)	0.011	2.42 (1.71–3.43)	<0.001
log-SII	1.14 (0.92–1.43)	0.237	1.53 (1.17–1.99)	0.002
(B) Multivariate Model (SIRI)				
log-SIRI	1.44 (1.13–1.83)	0.003	2.02 (1.53–2.67)	<0.001
Age	1.00 (0.98–1.02)	0.770	0.98 (0.96–1.00)	0.088
Albumin	0.86 (0.58–1.29)	0.472	0.40 (0.26–0.60)	<0.001
Prior lines	0.91 (0.81–1.04)	0.158	0.89 (0.77–1.01)	0.074
ECOG ≥ 2	1.51 (0.85–2.69)	0.160	1.26 (0.69–2.32)	0.458

(A) Comparative multivariable analysis: Each inflammatory marker (natural log-transformed) was analyzed in a separate multivariable model adjusted for the age, serum albumin, line of chemotherapy, and ECOG performance status. (B) Multivariable model: detailed results of the model using the log-SIRI as the representative inflammatory marker. Hazard ratios for continuous variables represent the effect per one-unit increase. Log-transformed values were used for inflammatory markers. Abbreviations: HR, hazard ratio; CI, confidence interval; PFS, progression-free survival; OS, overall survival; ECOG, Eastern Cooperative Oncology Group; SIRI, systemic inflammation response index; NLR, neutrophil-to-lymphocyte ratio; SII, systemic immune-inflammation index; PD-L1, programmed death-ligand 1.

## Data Availability

The raw data supporting the conclusions of this article will be made available by the authors upon request.

## Supplementary Materials

The following supporting information can be downloaded at: https://www.mdpi.com/article/10.3390/cancers18182905/s1, Figure S1: Distribution of peripheral blood inflammatory markers before and after natural log transformation; Table S1: Univariable Cox proportional hazards analysis for progression-free survival and overall survival.; Table S2: Sensitivity analyses of continuous log-SIRI; Table S3: Optimal cutoffs and discriminatory performance of inflammatory markers for identifying patients with durable disease control; Figure S2: Receiver operating characteristic (ROC) curves for inflammatory markers in patients with durable disease control to pembrolizumab monotherapy; Table S4: Sensitivity analysis of SIRI performance using alternative definitions of durable disease control (PFS ≥ 6 months vs. PFS ≥ 12 months); Table S5: Baseline characteristics according to the exploratory SIRI threshold; Table S6: Kaplan-Meier survival outcomes and restricted mean survival time (RMST) stratified by baseline SIRI.
