# Supplementary material for: Baseline Systemic Inflammation Response Index and Clinical Outcomes in Heavily Pretreated Platinum-Resistant Ovarian Cancer Treated with Pembrolizumab Monotherapy
Source: Cancers (Basel). 2026 Sep 8;18(18):2905. doi: 10.3390/cancers18182905 (PMC13604142; doi:10.3390/cancers18182905)
Supplement: Supplementary file 1 [file cancers-18-02905-s001.zip › cancers-4441697-supplementary.pdf]

Figure S1. Distribution of peripheral blood inflammatory markers before and after natural log transformation.

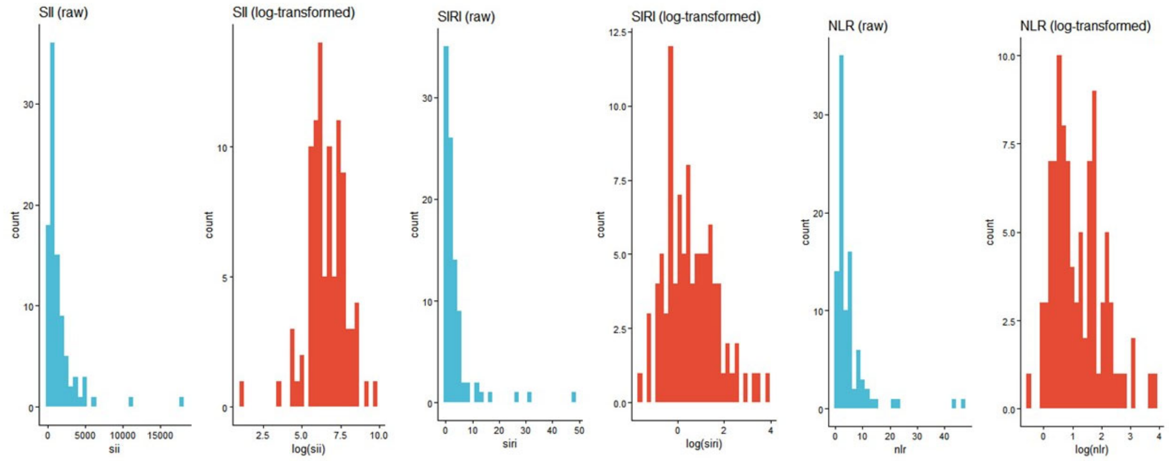

The histograms illustrate the frequency distribution of the Systemic Immune-Inflammation Index (SII), Systemic Inflammation Response Index (SIRI), and Neutrophil-to-Lymphocyte Ratio (NLR). The left panels (blue) display the raw values, exhibiting a distinct right-skewed distribution. The right panels (red) display the values after natural logarithmic transformation, demonstrating a reduction in skewness and an approximation to a normal distribution, which justified the use of log-transformed variables in the subsequent regression analyses.

Table S1**. Univariable Cox proportional hazards analysis for progression-free survival and overall survival.**

Variable	PFS HR (95%CI)	p-value	OS HR (95%CI)	p-value
Age	0.99 (0.98–1.01)	0.428	0.99 (0.97–1.01)	0.178
ECOG ≥2	1.74 (1.08–2.81)	0.023	2.32 (1.38–3.89)	0.001
Stage III–IV	1.07 (0.51–2.22)	0.860	1.34 (0.58–3.10)	0.487
High grade	1.27 (0.58–2.77)	0.545	3.04 (1.10–8.37)	0.032
Prior lines	0.93 (0.82–1.04)	0.197	0.88 (0.77–1.01)	0.075
Albumin	0.67 (0.47–0.95)	0.023	0.39 (0.27–0.56)	<0.001
Prior PARPi	1.30 (0.75–2.25)	0.349	1.77 (1.01–3.10)	0.047
Prior Bevacizumab	1.48 (0.94–2.34)	0.092	2.39 (1.40–4.08)	0.001
log-SIRI	**1.56 (1.24–1.97)**	**<0.001**	**2.18 (1.68–2.83)**	**<0.001**
log-NLR	**1.59 (1.20–2.10)**	**0.001**	**2.77 (2.01–3.82)**	**<0.001**
log-SII	1.19 (0.96–1.48)	0.117	**1.66 (1.29–2.14)**	**<0.001**

Hazard ratios for continuous variables (Age, Albumin, Prior lines, log-SIRI, log-NLR, log-SII) represent the effect per one-unit increase. Log-transformed values were used for inflammatory markers.

Abbreviations: HR, hazard ratio; CI, confidence interval; PFS, progression-free survival; OS, overall survival; ECOG, Eastern Cooperative Oncology Group; PARP, poly(ADP-ribose) polymerase; SIRI, systemic inflammation response index; NLR, neutrophil-to-lymphocyte ratio; SII, systemic immune-inflammation index.

Table S2. Sensitivity analyses of continuous log-SIRI

Analysis	PFS events	PFS HR for log-SIRI (95% CI)	p-value	OS events	OS HR for log-SIRI (95% CI)	p-value
**Primary model**	91	**1.44 (1.13–1.83)**	**0.003**	79	**2.02 (1.53–2.67)**	**<0.001**
Serous histology only (n=73)	71	**1.56 (1.15–2.11)**	**0.004**	61	**1.90 (1.39–2.58)**	**<0.001**
Baseline disease extent adjusted	91	**1.45 (1.12–1.87)**	**0.005**	79	**1.93 (1.44–2.59)**	**<0.001**
Prior bevacizumab/PARPi adjusted	91	**1.43 (1.13–1.83)**	**0.004**	79	**2.13 (1.60–2.82)**	**<0.001**
Prior treatment-course duration replacing prior lines	91	**1.41 (1.10–1.81)**	**0.007**	79	**1.93 (1.46–2.56)**	**<0.001**

Analysis	PFS events	PFS HR for log-SIRI (95% CI)	p-value	OS events	OS HR for log-SIRI (95% CI)	p-value
PD-L1 status adjusted	91	**1.44 (1.13–1.84)**	**0.003**	79	**2.01 (1.52–2.66)**	**<0.001**

The primary model was adjusted for age, serum albumin, number of prior treatment lines, and ECOG performance status. The disease-extent model additionally included radiographically evident peritoneal involvement, ascites, and log-transformed serum CA-125. Peritoneal involvement was defined using explicit evidence of active peritoneal seeding/carcinomatosis on imaging closest to pembrolizumab initiation (69/95 patients), and radiographically evident ascites was present in 29/95 patients. The prior-treatment-duration model replaced the number of prior treatment lines with the interval from initiation of first systemic treatment to pembrolizumab initiation because of strong collinearity between the two variables.

Table S3. Optimal cutoffs and discriminatory performance of inflammatory markers for identifying patients with durable disease control

Marker	Cutoff	AUC	Sensitivity	Specificity	PPV	NPV	Accuracy	Youden
**SIRI**	**1.15**	**0.727 (0.577–0.878)**	0.800	0.643	0.211	0.964	0.660	0.443
NLR	4.25	0.682 (0.513–0.851)	0.900	0.417	0.155	0.972	0.468	0.317
SII	996	0.653 (0.481–0.825)	0.900	0.440	0.161	0.974	0.489	0.340

Optimal cutoffs were determined using the Youden index. Cutoff values are back-transformed from the log scale to raw units.

One patient censored before 6 months without a PFS event was excluded. Thresholds and performance metrics were derived within the present cohort and were not externally validated.

Abbreviations: AUC, area under the curve; PPV, positive predictive value; NPV, negative predictive value; SIRI, systemic inflammation response index; NLR, neutrophil-to-lymphocyte ratio; SII, systemic immune-inflammation index.

Figure S2. Receiver operating characteristic (ROC) curves for inflammatory markers in patients with durable disease control to pembrolizumab monotherapy.

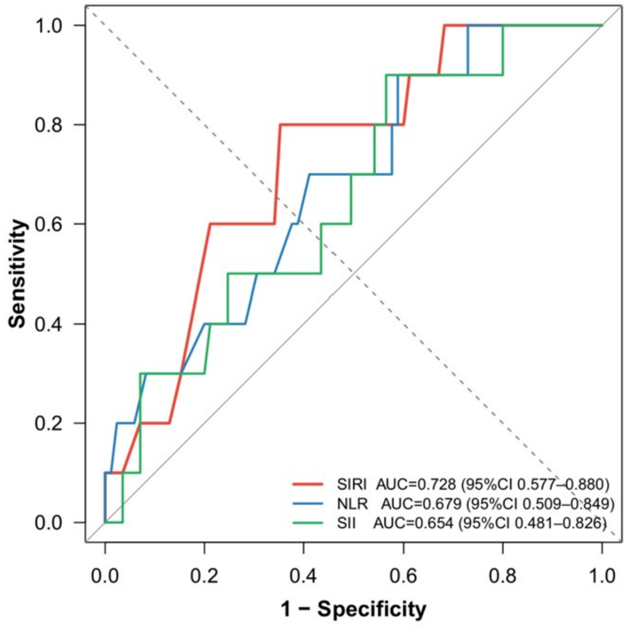

ROC curves for log-transformed SIRI, NLR, and SII as predictors of durable disease control (PFS ≥ 6 months) among 94 evaluable patients. The diagonal dashed line represents chance-level discrimination (AUC = 0.5). Optimal cutoffs were determined using the Youden index.

Abbreviations: ROC, receiver operating characteristic; AUC, area under the curve; CI, confidence interval; SIRI, systemic inflammation response index; NLR, neutrophil-to-lymphocyte ratio; SII, systemic immune-inflammation index; PFS,

progression-free survival.

Table S4. Sensitivity analysis of SIRI performance using alternative definitions of durable disease control (PFS ≥ 6months vs PFS ≥ 12 months).

	PFS≥6m (Primary)	PFS≥12m (Sensitivity)
Responder n (%)	10 (10.6%)	7 (7.4%)
Optimal SIRI cutoff	**1.15**	**1.15**
AUC (95%CI)	0.727 (0.577–0.878)	0.733 (0.575–0.891)
Sensitivity	0.800	0.857
Specificity	0.643	0.632
PPV	0.211	0.158
NPV	0.964	0.982
Youden index	0.443	0.489

The data-derived SIRI threshold remained 1.15 when durable disease control was alternatively defined as PFS ≥12 months. Cutoff values are back-transformed from the log scale to raw units. DeLong test was used to compare AUCs between the two definitions.

Abbreviations: AUC, area under the curve; CI, confidence interval; PPV, positive predictive value; NPV, negative predictive value; SIRI, systemic inflammation response index; PFS, progression-free survival.

Table S5. Baseline characteristics according to the exploratory SIRI threshold

**Characteristic**	**Low SIRI ≤1.15 (n=38)**	**High SIRI >1.15 (n=57)**	**p-value**
Age, years, mean (SD)	56.1 (9.9)	56.0 (11.4)	0.975
Age ≥65 years	6 (15.8%)	12 (21.1%)	0.600
ECOG ≥2	5 (13.2%)	18 (31.6%)	0.051
**Histologic type**			0.075
Serous carcinoma	33 (86.8%)	40 (70.2%)	
Clear cell carcinoma	2 (5.3%)	12 (21.1%)	
Other	3 (7.9%)	5 (8.8%)	
High grade	34 (89.5%)	54 (94.7%)	0.432
Optimal primary surgery	16 (42.1%)	23 (40.4%)	1.000
Initial FIGO stage III–IV	35 (92.1%)	52 (91.2%)	1.000
**Number of prior systemic treatment lines**			0.076
1	0 (0.0%)	4 (7.0%)	
2–3	15 (39.5%)	30 (52.6%)	
≥4	23 (60.5%)	23 (40.4%)	
Time from initiation of first systemic treatment to pembrolizumab, months, median (IQR)	**37.4 (19.3–71.2)**	**24.0 (14.2–43.2)**	**0.019**
Serum CA-125, U/mL, median (IQR)	211.5 (96.5–519.5)	417.0 (89.0–919.0)	0.130
Radiographically evident ascites	8 (21.1%)	21 (36.8%)	0.117
Definite active peritoneal involvement	28 (73.7%)	41 (71.9%)	1.000
Albumin, g/dL, median (IQR)	**4.3 (3.9–4.5)**	**3.9 (3.3–4.2)**	**0.003**
PD-L1 positive	34 (89.5%)	53 (93.0%)	0.709
dMMR, n/N available (%)	6/19 (31.6%)	11/29 (37.9%)	0.762

Characteristic	Low SIRI ≤1.15 (n=38)	High SIRI >1.15 (n=57)	p-value
BRCA mutation, n/N available (%)	8/37 (21.6%)	10/50 (20.0%)	1.000
Prior PARP inhibitor use	8 (21.1%)	8 (14.0%)	0.410
Prior bevacizumab use	23 (60.5%)	43 (75.4%)	0.172

Continuous variables were compared using Welch's t-test or the Mann–Whitney U test, as appropriate, and categorical variables using Fisher's exact test. The SIRI threshold of 1.15 was data-derived and exploratory. MMR and BRCA percentages were calculated among patients with available data.

Table S6. Kaplan-Meier survival outcomes and restricted mean survival time (RMST) stratified by baseline SIRI.

Outcome	Low SIRI (n=38)	High SIRI (n=57)	p-value
**Objective response rate, n (%)**	**7 (18.4)**	**1 (1.8)**	**0.006**
**Disease control rate, n (%)**	**13 (34.2)**	**11 (19.3)**	**0.148**
**Progression-Free Survival (PFS)**			
Median PFS, months (95% CI)	2	2	0.013
6-month PFS rate	21.1%	**1.9%**	
12-month PFS rate	15.8%	**1.9%**	
24-month PFS rate	10.5%	**1.9%**	
12-month RMST, months (95% CI)	3.97 (2.76-5.19)	2.16 (1.68–2.65)	0.007
**Overall Survival (OS)**			
Median OS, months (95% CI)	16 (11-23)	4 (3-5)	<0.001
6-month OS rate	78.9%	34.7%	
12-month OS rate	52.6%	17.0%	
24-month OS rate	36.8%	3.0%	
24-month RMST, months (95% CI)	14.42 (11.63–17.21)	6.75 (5.01–8.48)	<0.001

Patients were dichotomized into low SIRI (≤ 1.15) and high SIRI (> 1.15) groups based on the Youden-optimal cutoff. Survival rates were estimated using the Kaplan-Meier method. RMST was calculated at prespecified time horizons (12 months for PFS; 24 months for OS). Differences in RMST between groups were compared using the two-sample RMST test. p-values from the log-rank test. ORR and DCR were compared using Fisher's exact test.

Abbreviations: SIRI, systemic inflammation response index; PFS, progression-free survival; OS, overall survival; CI, confidence interval; RMST, restricted mean survival time.
